# Draft Genome Sequence of *Klebsiella pneumoniae* KGM-IMP216 Harboring *bla*_CTX-M-15_, *bla*_DHA-1_, *bla*_TEM-1B_, *bla*_NDM-1_, *bla*_SHV-28_, and *bla*_OXA-1_, Isolated from a Patient in Lebanon

**DOI:** 10.1128/genomeA.01632-15

**Published:** 2016-01-28

**Authors:** Sima Tokajian, Jonathan A. Eisen, Guillaume Jospin, Ghassan Matar, George F. Araj, David A. Coil

**Affiliations:** a Department of Natural Sciences, School of Arts and Sciences, Lebanese American University, Byblos, Lebanon; b University of California Davis Genome Center, Davis, California, USA; c Department of Experimental Pathology, Immunology and Microbiology, Faculty of Medicine, American University of Beirut, Beirut, Lebanon; d Department of Pathology and Laboratory Medicine, Faculty of Medicine, American University of Beirut, Beirut, Lebanon

## Abstract

We present the draft genome of highly drug-resistant *Klebsiella pneumoniae* KGM-IMP216, isolated from a urine sample collected from a patient in Lebanon. The draft genome sequence consisted of 77 contigs, including a combined 5,731,500 bases with 57% G+C content.

## GENOME ANNOUNCEMENT

*Klebsiella pneumoniae* is among the most important causes of both hospital and community-acquired serious bacterial infections in humans. Extended-spectrum β-lactamase (ESBL)–producing organisms remain important causes of the failure of therapy with cephalosporins and have serious infection-control consequences. Infections due to ESBL-producing *K. pneumoniae* are most often involved in urinary and respiratory tract infections (1). The ESBL genes are mostly plasmid-encoded (2), and most ESBLs can be divided into three genotypes: TEM, SHV, and CTX-M (3). The emergence of ESBL-producing bacteria is now a critical concern. β-lactamases are able to hydrolyze oxyimino-cephalosporins and aztreonam, and constitute an increasingly important mechanism of antimicrobial resistance among nosocomial Gram-negative pathogens (4). ESBLs of the CTX-M type, with CTX-M-15 being the variant most commonly identified in species of human origin, and the plasmid-encoded AmpC-type β-lactamases (*bla*_DHA_) have been recognized to be of growing importance (5). Carbapenems are the primary therapeutic agents for infections caused by ESBLs. The extensive use of carbapenems led to the emergence of isolates with resistance genes coding for carbapenemases (6). Among the clinically significant carbapenemases are class B (NDM-1: New Delhi metallo-β-lactamase-1) and class D (OXA-1) (7).

In this study we sequenced *K. pneumoniae* KGM-IMP216 of multilocus sequence type ST-14 harboring *bla*_CTX-M-15_, *bla*_DHA-1_, *bla*_TEM-1B_, *bla*_NDM-1_, *bla*_SHV-28_, and *bla*_OXA-1_*,* isolated from a urine sample at the American University of Beirut Medical Center in Lebanon (8–10).

A Nextera XT kit (Illumina, San Diego, CA, USA) was used to simultaneously fragment and adapter-tag the library, as per the manufacturer’s instructions. The library was normalized by bead-based affinity and then sequenced using the MiSeq version 3 600-cycle kit (Illumina) to perform 300-bp paired-end sequencing on a MiSeq instrument (Illumina), per the manufacturer’s instructions. Quality trimming and error correction of the reads resulted in 4,192,299 high-quality reads. All sequence processing and assembly was performed using the A5-miseq assembly pipeline. This pipeline automates the processes of data cleaning, error correction, contig assembly, scaffolding, and quality control (11, 12). The initial assembly produced 77 contigs, for which no scaffolding was obtained. The final collection of contigs was submitted to GenBank. The final draft genome sequence consisted of a combined 5,731,500 bases with 57% G+C content. Automated annotation was performed using the RAST annotation server (13). *K. pneumoniae* KGM-IMP216 contains 5,499 predicted coding sequences and 112 predicted RNAs. Moreover, IncN, IncL/M, IncFII(K), IncFIA(HI1), IncR, and IncFIB(K) plasmid types were detected using the PlasmidFinder version 1.3 web server (https://cge.cbs.dtu.dk/services/PlasmidFinder) (14).

### Nucleotide sequence accession numbers.

This whole-genome shotgun project has been deposited at DDBJ/EMBL/GenBank under the accession number LJOI00000000. The version described in this paper is the first version, LJOI01000000.

## References

[1] Paterson DL, Bonomo RA 2005 Extended-spectrum β-lactamases: a clinical update. Clin Microbiol Rev 18:657–686. doi:10.1128/CMR.18.4.657-686.2005. 16223952 PMC1265908

[2] Nakamura T, Komatsu M, Yamasaki K, Fukuda S, Miyamoto Y, Higuchi T, Ono T, Suevoshi N, Kida K, Toda H, Tovokawa M, Nishi I, Sakamoto M, Akagi M, Nakai I, Kofuku T, Orita T, Wada Y, Zikimoto T, Koike C, Kinoshita S, Hirai I, Takahashi H, Matsuura N, Yamamoto Y 2012 Epidemiology of *Escherichia coli*, *Klebsiella* species, and *Proteus mirabilis* strains producing extended-spectrum β-lactamases from clinical samples in the Kinki region of Japan. Am J Clin Pathol 137:620–6266. doi:10.1309/AJCP48PDVKWQOXEZ. 22431539

[3] Paterson L 2006 Resistance in gram-negative bacteria: *Enterobacteriaceae*. Am J Infect Control 34(suppl 1):S20–S28, S64–S73. doi:10.1016/j.ajic.2006.05.238. 16813978

[4] Jacoby GA 1997 Extended-spectrum β-lactamases and other enzymes providing resistance to oxyimino-β-lactams. Infect Dis Clin North Am 11:875–887. doi:10.1016/S0891-5520(05)70395-0. 9421705

[5] Schluter A, Nordmann P, Bonnin RA, Millemann Y, Eikmeyer FG, Wibberg D, Puhler A, Poirel L 2014 IncH-type plasmid harboring *bla*_CTX-M-15_, *bla*_DHA-1_, and *qnrB4* genes recovered from animal isolates. Antimicrob Agents Chemother 58:3768–3773. doi:10.1128/AAC.02695-14. 24752252 PMC4068538

[6] Nordmann P, Naas T, Poirel L 2011 Global spread of carbapenemase-producing *Enterobacteriaceae*. Emerg Infect Dis 17:1791–1798. doi:10.3201/eid1710.110655. 22000347 PMC3310682

[7] Barantsevich E, Churkina I, Barantsevich N, Schlyakhto E, Woodford N 2013 Emergence of *Klebsiella pneumoniae* producing NDM-1 carbapenemase in Saint Petersburg, Russia. J Antimicrob Chemother 68:1204–1206. doi:10.1093/jac/dks503. 23315490

[8] Baroud M, Dandache I, Araj GF, Wakim R, Kanj S, Kanafani Z, Khairallah M, Sabra A, Shehab M, Dbaibo G, Matar GM 2013 Underlying mechanisms of carbapenem resistance in extended-spectrum β-lactamase-producing *Klebsiella pneumoniae* and *Escherichia coli* isolates at a tertiary care centre in Lebanon: role of OXA-48 and NDM-1 carbapenemases. Int J Antimicrob Agents 41:75–79. doi:10.1016/j.ijantimicag.2012.08.010. 23142087

[9] El-Herte RI, Araj GF, Matar GM, Baroud M, Kanafani ZA, Kanj SS 2012 Detection of carbapenem-resistant *Escherichia coli* and *Klebsiella pneumoniae* producing NDM-1 in Lebanon. J Infect Dev Ctries 6:457–461. doi:10.3855/jidc.2340. 22610714

[10] El-Herte RI, Kanj SS, Matar GM, Araj GF 2012 The threat of carbapenem-resistant *Enterobacteriaceae* in Lebanon: an update on the regional and local epidemiology. J Infect Public Health 5:233–243. doi:10.1016/j.jiph.2012.02.003. 22632597

[11] Tritt A, Eisen JA, Facciotti MT, Darling AE 2012 An integrated pipeline for de novo assembly of microbial genomes. PLoS One 7:e42304 doi:10.1371/journal.pone.0042304. 23028432 PMC3441570

[12] Coil D, Jospin G, Darling AE 2015 A5-miseq: an updated pipeline to assemble microbial genomes from Illumina MiSeq data. Bioinformatics 31:587–589. doi:10.1093/bioinformatics/btu661. 25338718

[13] Aziz RK, Bartels D, Best AA, DeJongh M, Disz T, Edwards RA, Formsma K, Gerdes S, Glass EM, Kubal M, Meyer F, Olsen GJ, Olson R, Osterman AL, Overbeek RA, McNeil LK, Paarmann D, Paczian T, Parrello B, Pusch GD, Reich C, Stevens R, Vassieva O, Vonstein V, Wilke A, Zagnitko O 2008 The RAST server: Rapid Annotations using Subsystems Technology. BMC Genomics 9:75. doi:10.1186/1471-2164-9-75. 18261238 PMC2265698

[14] Carattoli A, Zankari E, Garcia-Fernandez A, Voldby Larsen M, Lund O, Villa L, Møller Aarestrup F, Hasman H 2014 *In silico* detection and typing of plasmids using PlasmidFinder and plasmid multilocus sequence typing. Antimicrob Agents Chemother 58:3895–3903. doi:10.1128/AAC.02412-14. 24777092 PMC4068535

